# Usefulness of Soluble Transferrin Receptor in the Diagnosis of Iron Deficiency Anemia in Rheumatoid Arthritis Patients in Clinical Practice

**DOI:** 10.1155/2022/7067262

**Published:** 2022-10-12

**Authors:** Florian Günther, Rainer H. Straub, Wolfgang Hartung, Martin Fleck, Boris Ehrenstein, Louisa Schminke

**Affiliations:** ^1^Department of Rheumatology and Clinical Immunology, Asklepios Clinic, Bad Abbach, Germany; ^2^Department of Internal Medicine I, University Medical Center, Regensburg, Germany

## Abstract

**Aim:**

We analyzed the added value of sTfR measurement in routine clinical practice to standard parameters (SP) of iron deficiency in the detection of iron deficiency anemia (IDA) in patients with rheumatoid arthritis (RA).

**Methods:**

Blood samples from 116 patients with RA were analyzed in a prospective study. Based on biochemical parameters, patients were classified as having IDA, anemia of chronic disease (ACD), IDA with concomitant ACD (ACD/IDA), or “other anemia.” Sensitivity, specificity, positive (PPV), and negative predictive values (NPV) of sTfR and SP of iron status alone and in combination were calculated for the diagnosis of IDA in general, i.e., IDA or ACD/IDA.

**Results:**

In the whole sample, with regard to the diagnosis of iron deficiency (IDA or ACD/IDA), sTfR had a higher sensitivity compared both to the combined use of SP and to the combination of SP with sTfR (80.9% versus 66.7/54.8%). Specificity, PPV and NPV did not differ substantially. When patients were stratified in groups with high (CRP levels above the median, i.e., 24.1 mg/l) and low (CRP levels less or equal to the median) inflammation, the diagnostic superiority of sTfR was restricted to patients with high inflammation. In this group, the diagnostic performance of sTfR was superior both to the combined use of SP and the combination of SP with sTfR with higher sensitivity (100% versus 52.4%) and NPV (100% versus 77.7/76.7%) and comparable specificity and PPV.

**Conclusion:**

For the detection of iron depletion (IDA or ACD/IDA) in anemic RA patients, sTfR is superior to SP of iron deficiency only in highly inflammatory states.

## 1. Introduction

Despite decreasing time trends in the era of biological disease modifying drugs, anemia is one of the most common extra-articular manifestations of patients suffering from rheumatoid arthritis (RA) with a reported prevalence between 15 and 60% [1–3]. It is well established that anemia in RA is related to higher disease activity, worse outcome parameters, and increased mortality [4–6]. Different groups demonstrated anemia to predict radiographic damage in RA [1, 3, 7].

As anemia in RA is mostly considered as the prototypical type of anemia of chronic disease (ACD) or anemia of inflammation (AI)—the terms are used interchangeably—the diagnosis of anemia in RA should always be followed by a thorough search for (subclinical) disease activity. However, it is pivotal to identify other or coexisting frequent causes of anemia in RA. Whereas the decisive treatment for ACD in systemic rheumatic diseases is remission of the underlying disease [8], anemia in RA due to iron deficiency, vitamin deficiency, or treatment-related anemia requires different diagnostic and therapeutic interventions.

Patients with chronic inflammatory disease frequently suffer from a combination of ACD and iron deficiency anemia (IDA). In RA, it is estimated that iron deficiency contributes to anemia prevalence in 30–50% of cases [1]. In the absence of inflammation, serum ferritin as an indicator of total body iron stores is the most useful parameter to differentiate ACD from IDA [9–11]. While a reduction in serum ferritin below 30 ng/ml shows absolute or true iron deficiency with high diagnostic accuracy (sensitivity 92%, specificity 98%), patients with ACD present with normal or increased ferritin levels [2, 12]. However, in acute and chronic inflammatory disorders, high concentrations of serum ferritin result from increased secretion by iron-retaining macrophages. Furthermore, serum ferritin is an acute-phase protein that is induced by inflammatory mediators [2, 9]. Thus, in inflammatory states, ferritin loses its diagnostic value as an indicator of total iron body stores.

The main challenge in ACD is to identify patients with concomitant true iron deficiency, as these patients need specific evaluation for gastrointestinal blood loss and iron-targeted management strategies. Several biomarkers have been studied for their potential to detect iron deficiency in the presence of inflammation. Among these, the soluble transferrin receptor (sTfR) is the biomarker that is most frequently used in clinical routine.

Serum levels of sTfR have been shown to differentiate effectively between IDA, in which sTfR is usually increased and ACD [13–16]. Although inflammation negatively affects the sensitivity of sTfR to indicate true iron deficiency [2, 9, 16], sTfR levels tend to be normal in ACD. Therefore, elevated sTfR levels in the setting of ACD suggest the presence of additional absolute ID [14–17].

In the present study, we analyzed the added value of sTfR measurement in routine clinical practice to standard parameters of iron deficiency in the detection of IDA alone or concomitant IDA and ACD in anemic patients with rheumatoid arthritis as a prototype of chronic, autoimmune inflammatory disease.

## 2. Material and Methods

### 2.1. Patient Characteristics

The study population consisted of 116 anemic in- and outpatients suffering from rheumatoid arthritis (RA), which consecutively attended our tertiary rheumatology center between December 2019 and December 2020. The local ethics committee of the University Regensburg approved the study (approval number 12-101-0074), and written informed consent was obtained from all participants. All patients fulfilled the 2010 ACR/EULAR classification criteria for RA [18].

Anemia was defined following the reference ranges set by the manufacturer of the cell counter used by the central laboratory of the Asklepios Clinic for Rheumatology/Clinical Immunology in Bad Abbach (XN1000-analyzer, Sysmex, Norderstedt, Germany) as a hemoglobin concentration of <12 g/dl for woman and <14 g/dl for men.

Subject exclusion criteria were hemolytic anemia, blood transfusions within the past three months, trauma-associated bleeding, hematologic malignancies, cancer patients currently receiving chemotherapy or who received chemotherapy within the last 6 months, renal failure patients currently on dialysis, vitamin B12 or folate deficiency, and patients currently taking iron supplements or receiving recombinant erythropoietin.

### 2.2. Classification of Anemia

Anemia in RA patients was classified as IDA, ACD, or a combination of the two (ACD/IDA) following previously described algorithms [19–21]. Specifically, patients with anemia were classified as having IDA if active inflammation (defined as a CRP level of >10 mg/l or an ESR of ≥30 mm/hour) was absent, and at least 1 of the following 2 conditions was met: (1) transferrin saturation (TSAT) < 20% and ferritin level < 30 ng/ml; (2) sTfR index (sTfR divided by log − transformed ferritin values) ≥ 3.2. Patients were classified as having ACD if active inflammation was present, and at least 1 of the following 2 conditions was met: (1) TSAT < 20% and ferritin level ≥ 100 *μ*g/ml; (2) sTfR index (sTfR divided by log − transformed ferritin values) < 2 and ferritin level ≥ 30 ng/ml. Patients were classified as having ACD/IDA if active inflammation was present, and at least 1 of the following 2 conditions was met: (1) TSAT < 20% and ferritin level < 100 ng/ml; (2) sTfR index (sTfR divided by log − transformed ferritin values) ≥ 2. Patients that could not be classified according to these definitions were categorized as having “other anemia.”

### 2.3. Sensitivity and Specificity of sTfR and Standard Parameters of Iron Status

As the primary clinical utility of sTfR is to identify IDA in general, with or without accompanying ACD, sensitivity, specificity, PPV, and NPV were calculated for the identification of IDA or ACD/IDA. In this context, sensitivity is defined as the percentage of patients with IDA or ACD/IDA correctly identified. Specificity is defined by the percentage of patients with ACD (without accompanying IDA) or “other anemia” correctly identified.

Standard parameters of iron status, CRP levels, and sTfR were stepwise combined: after calculation of the diagnostic performance (sensitivity, specificity, PPV, and NPV) of (1) red blood cell indices (RBCI) alone and (2) serum ferritin alone, sensitivity, specificity, PPV, and NPV of the combination of (3) serum ferritin and RBCI, the combination of (4) serum ferritin, TSAT and RBCI, the combination of (5) serum ferritin, TSAT, CRP level, and RBCI, and, in a last step, the combination of (6) serum ferritin, TSAT, CRP level, sTfR, and RBCI were calculated.

Correct identification of IDA by sTfR and by standard parameters of iron status alone and in combination were defined as follows:

- sTfR: levels > 5.0 mg/l for men and >4.0 mg/l for women, according to the reference ranges set by the manufacturer of the sTfR assay (Roche Diagnostics, Mannheim, Germany)
- RBCI: presence of both microcytosis (mean corpuscular volume < 82 fl) and hypochromasia (mean corpuscular hemoglobin < 28 pg or mean corpuscular hemoglobin concentration < 32 g/dl)
- Serum ferritin: levels < 30 ng/ml
- Serum ferritin and RBCI: serum ferritin levels < 30 ng/ml or the presence of both microcytosis and hypochromasia
- Serum ferritin, TSAT, and RBCI: ferritin levels < 30 ng/ml concurrent with a TSAT < 20% or the presence of both microcytosis and hypochromasia
- Serum ferritin, TSAT, CRP, and RBCI: ferritin levels < 30 ng/ml concurrant with a TSAT < 20%, a CRP − level < 10 mg/l or the presence of both microcytosis and hypochromasia
- Serum ferritin, TSAT, CRP, sTfR, and RBCI: ferritin levels < 30 ng/ml concurrent with a TSAT < 20%, a CRP − level < 10 mg/l and increased sTfR (>5.0 mg/l for men and >4.0 mg/l for women) or the presence of both microcytosis and hypochromasia

Correct identification of ACD/IDA by sTfR and by standard parameters of iron status alone and in combination were defined as follows:

- sTfR: levels > 5.0 mg/l for men and >4.0 mg/l for women, according to the reference ranges set by the manufacturer of the sTfR assay (Roche Diagnostics, Mannheim, Germany)
- RBCI: presence of both microcytosis (mean corpuscular volume < 82 fl) and hypochromasia (mean corpuscular hemoglobin < 28 pg or mean corpuscular hemoglobin concentration < 32 g/dl)
- Serum ferritin: levels ≥ 30 ng/ml and <100 ng/ml
- Serum ferritin and RBCI: serum ferritin levels ≥ 30 ng/ml and <100 ng/ml or the presence of both microcytosis and hypochromasia
- Serum ferritin, TSAT, and RBCI: serum ferritin levels ≥ 30 ng/ml and <100 ng/ml concurrent with a TSAT < 20% or the presence of both microcytosis and hypochromasia
- Serum ferritin, TSAT, CRP, and RBCI: serum ferritin levels ≥ 30 ng/ml and <100 ng/ml concurrent with a TSAT < 20% and a CRP − level ≥ 10 mg/l or the presence of both microcytosis and hypochromasia
- Serum ferritin, TSAT, CRP, sTfR, and RBCI: ferritin levels ≥ 30 ng/ml and <100 ng/ml concurrent with a TSAT < 20%, a CRP − level ≥ 10 mg/l and increased sTfR (>5.0 mg/l for men and >4.0 mg/l for women) or the presence of both microcytosis and hypochromasia

### 2.4. Stratification of Patients

To perform a differentiated evaluation of the added value of sTfR dependent on the inflammatory activity of the rheumatic disease, patients were stratified in subjects with low and high inflammatory activity. Low inflammatory activity was operationalized as serum CRP levels less or equal to the median of serum CRP (24.1 mg/l). High inflammation was defined as a CRP level above the median.

### 2.5. Laboratory Analysis

Nonfasting blood samples were collected in the morning. sTfR serum levels were determined using a commercial particle enhanced immunoturbidimetric assay (Tina-quant Soluble Transferrin Receptor II, Roche Diagnostics, Mannheim, Germany). The assay was performed on a Cobas c 501 analyzer (Roche Diagnostics, Mannheim, Germany). The limit of detection (LOD) of the test was 0.40 mg/l (4.72 nmol/l). The average intra- and interassay coefficient of variation was 1.5 and 1.7%, respectively. Serum ferritin levels were measured using a commercial particle enhanced immunoturbidimetric assay (Tina-quant Ferritin Gen 4, Roche Diagnostics, Mannheim, Germany) performed on a Cobas c 501 analyzer (Roche Diagnostics, Mannheim, Germany). Iron, plasma transferrin concentration, and CRP were measured on a Cobas c 501 analyzer (Roche Diagnostics, Mannheim, Germany; iron: colorimetric assay; transferrin and CRP: immunoturbidimetric assay). The percent plasma transferrin saturation was calculated using the following formula: serum − iron [*μ*g/dl]/serum − transferrin [mg/dl] × 70.9. Blood counts were measured with an automated hematology analyzer (XN1000-analyzer, Sysmex, Norderstedt, Germany). The ESR was determined by Westergren method using an SRS 100/II analyzer (Electa-Lab S.r.l., Forli, Italy).

### 2.6. Statistical Analysis

Results were analyzed using the Statistical Package for Social Sciences for Windows, version 25.0 (SPSS, Chicago, IL, USA). Correlations between sTfR and traditional parameters of iron metabolism, inflammatory markers, and DAS 28 were analyzed using Spearman's rho correlation analysis. Correlation coefficients between 0 and 0.3 indicate a weak positive relationship, values between 0.3 and 0.7, and a moderate positive relationship. Values between 0.7 and 1.0 indicate a strong positive linear relationship. Following Kruskal-Wallis one-way analysis of variance on ranks, the distribution of sTfR values by anemia classification was evaluated by pairwise multiple comparison procedures (Dunn's method). Receiver-operating characteristic (ROC) curves and the area under the curve (AUC) were used to evaluate and compare the discriminatory ability of sTfR and ferritin in the diagnosis of iron deficiency, i.e., IDA or ACD/IDA. The AUC summarizes the diagnostic accuracy of the test. The AUC value lies between 0 and 1, and the closer the value is to 1, the better the test is. An AUC with a value of 0.5 suggests no discrimination. 95% confidence intervals of AUC values were used to assess statistically significant differences between AUC values. Lack of overlap between confidence intervals defined statistically significant differences. A *p* value of <0.05 was considered significant.

## 3. Results

### 3.1. Characteristics of Patients

Serum samples of 116 patients fulfilling the 2010 ACR/EULAR classification criteria for RA were analyzed for sTfR and standard parameters of iron deficiency. Characteristics of patients under study are displayed in Table 1.

### 3.2. Correlation of sTfR with Standard Parameters of Iron Metabolism, Inflammatory Markers, and DAS 28

Moderate correlations between sTfR and standard markers of iron deficiency, i.e., hemoglobin, ferritin, iron, TSAT, MCH, MCHC, and MCV, were determined as depicted in Table S1 (Supplementary).

Both sTfR and standard parameters of iron deficiency correlated significantly with inflammatory markers (CRP, ESR) and RA activity index (DAS 28). sTfR and red cell blood indices demonstrated weak, serum iron concentration, transferrin, TSAT, and ferritin moderate correlations with inflammatory markers and DAS 2 (Table S2, Supplementary).

### 3.3. Distribution of sTfR Values by Anemia Classification

**Figure 1:**
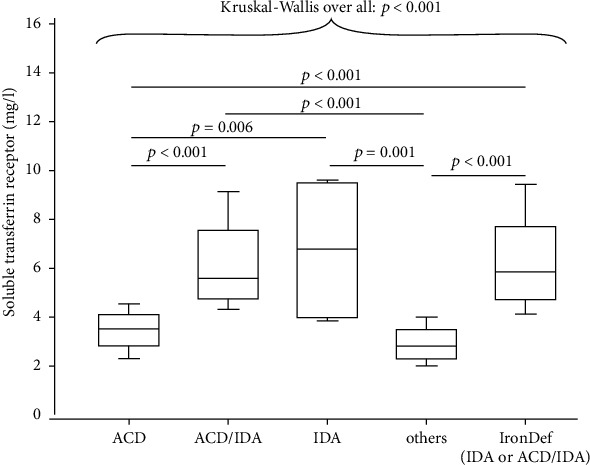
Serum levels of soluble transferrin receptor in anemic patients with RA demonstrated as box plots with the 90th, 75th, 50th (median), 25th, and 10^th^ percentile. IDA: iron deficiency anemia; ACD: anemia of chronic disease.

The distribution of sTfR values by anemia classification is shown in Figure 1. Serum sTfR levels in patients with IDA or ACD/IDA were significantly elevated compared to patients with ACD. Furthermore, a clear separation of patients with IDA or ACD/IDA from those with “other anemia” was observed.

Sensitivity, specificity, and predictive values of sTfR and standard markers of iron status.

The diagnostic performance of sTfR and traditional parameters of iron status alone and in combination were evaluated calculating the diagnostic sensitivity, specificity, and positive and negative predictive values in the diagnosis of IDA with or without accompanying ACD, i.e., IDA or ACD/IDA.

### 3.4. Diagnosis of Iron Deficiency, i.e., IDA or ACD/IDA

In the whole sample, with regard to the diagnosis of iron deficiency in general, i.e., IDA or ACD/IDA, the use of sTfR alone results in a higher sensitivity compared to the combination of standard parameters (80.9 versus 66.7%). The combination of standard parameters with sTfR does not increase sensitivity. Specificity, PPV, and NPV of sTfR alone, combined standard parameters, and the combination of traditional parameters with sTfR were comparable (Table 2).

In contrast to the results for the whole sample, in patients with low inflammatory activity, i.e., CRP-levels below the median (24.1 mg/l), the combined use of standard parameters of iron deficiency resulted in a higher sensitivity compared both to the use of sTfR alone (76.2 versus 66.7%) and to the combination of standard parameters with sTfR (76.2% versus 57.1%; Table 3). Specificity, PPV, and NPV did not differ substantially.

In contrast to the results in patients with low inflammatory activity, in patients with high inflammatory activity (CRP-levels above the median, i.e., 24.1 mg/l), the diagnostic performance of sTfR alone was highly superior both to the combination of standard parameters of iron deficiency and the combination of traditional markers of iron status with sTfR with higher sensitivity (100% versus 52.4%) and NPV (100% versus 77.7% and 76.7%, respectively) and comparable specificity and PPV (Table 4).

### 3.5. Discriminatory Ability of sTfR in the Diagnosis of Iron Deficiency

**Figure 2:**
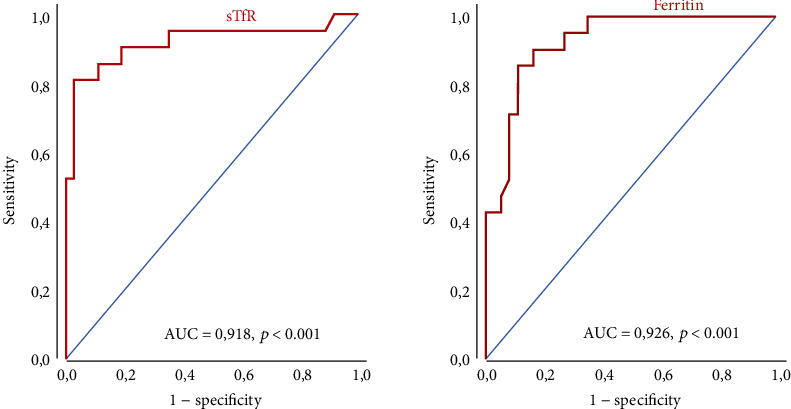
ROC curves for sTfR and ferritin in the diagnosis of iron deficiency, i.e., IDA or ACD/IDA, in patients with rheumatoid arthritis with low inflammatory activity. IDA: iron deficiency anemia; ACD: anemia of chronic disease.

Receiver-operating characteristic (ROC) curves were used to evaluate and compare the discriminatory ability of sTfR and ferritin in the diagnosis of iron deficiency, i.e., IDA or ACD/IDA. Compared to sTfR, there was no substantial difference in the discriminatory ability of ferritin to identify iron deficiency (i.e., IDA or ACD/IDA) in patients with low inflammatory activity (CRP levels less or equal to the median of serum CRP, i.e., 24.1 mg/l; Figure 2). In patients with high inflammatory activity (CRP level above the median) compared to sTfR, the discriminatory ability of ferritin was slightly lower but failed to reach statistical significance (AUC_sTfR_ 0.97 versus AUC_Ferritin_ 0.91).

## 4. Discussion

While ACD is the prototypical type of anemia in chronic autoimmune inflammatory diseases, the detection of IDA alone or concomitant ACD and IDA in inflammatory diseases is pivotal requiring different diagnostic and therapeutic interventions. As circulating ferritin, the “landmark” indicator for body iron stores, is positively influenced by inflammation [2, 9], the diagnosis of IDA or ACD with concomitant true iron deficiency (ACD/IDA) in inflammatory states is challenging.

Among several markers studied for their potential to detect true iron deficiency in inflammatory states, sTfR is the most frequently used biomarker in clinical routine considered to be unaffected by inflammation [12, 20, 22]. Despite the importance of a precise differential diagnosis between IDA, ACD, and a combination of both forms because of differing treatment and diagnostic strategies, there is a lack of data defining the position of sTfR in the diagnostic algorithm of IDA or ACD/IDA in routine clinical practice and the added value of sTfR to traditional parameters of iron deficiency in inflammatory diseases.

To our knowledge, this is the first study evaluating the added value of sTfR measurement in routine clinical practice compared to traditional parameters of iron deficiency alone and in combination in the differential diagnosis of anemia in patients with RA as a prototype of chronic autoimmune inflammatory disease.

In the present study, we found significantly elevated serum sTfR levels in patients with IDA or ADC/IDA compared to patients with ACD or “other anemia.” These results are consistent with previously published studies demonstrating that sTfR is a useful biomarker to detect iron-depleted anemic states and to differentiate effectively between IDA and ACD/IDA on the one hand and ACD on the other hand [13, 14, 23, 24].

To evaluate the diagnostic performance of sTfR and standard parameters of iron status alone and in combination, sensitivity, specificity, negative, and positive predictive values in the diagnosis of iron deficiency, i.e., IDA or ACD/IDA, were calculated. We found that with regard to the detection of iron deficiency in general, i.e., IDA or ACD/IDA, the use of sTfR alone resulted in a higher sensitivity (with similar specificity, PPV and NPV) compared to the combination of standard parameters of iron deficiency. Combination of standard parameters of iron deficiency and sTfR did not increase sensitivity, specificity, PPV, and NPV.

However, this diagnostic superiority of sTfR was restricted to patients with high inflammatory activity. In patients with low inflammation, the combination of standard parameters of iron status yielded an even higher sensitivity in comparison both to the use of sTfR alone and to the combination of standard parameters with sTfR. Specificity, PPV, and NPV did not differ substantially.

The finding, that, in patients with low inflammatory activity, the measurement of sTfR did not result in a higher diagnostic accuracy was reflected in ROC curve analysis which demonstrated that in patients with low inflammation, the discriminatory ability of sTfR and ferritin to identify iron deficiency did not differ substantially. Beyond that, superior diagnostic performance of sTfR in the diagnosis of iron deficiency in patients with high inflammatory activity was supported by ROC curve analysis which showed a slightly higher discriminatory ability of sTfR compared to ferritin in the diagnosis of iron deficiency in patients with high inflammation. However, comparison of AUC values failed to reach statistical significance.

We suggest that the superior diagnostic performance of sTfR in the diagnosis of iron deficiency in highly inflammatory states is due to our finding that, in comparison to standard parameters of iron status, with the exception of red cell blood indices, sTfR is least influenced by inflammatory activity operationalized as CRP- and ESR-levels.

While several studies demonstrated that serum levels of sTfR differentiate effectively between IDA and ACD [13–15, 25], the number of studies evaluating the diagnostic performance of sTfR compared to standard parameters of iron deficiency is limited.

Pettersson et al. found that, in 34 patients with a chronic rheumatic disease, the determination of sTfR did not prove superior to serum ferritin both in the distinction between IDA and ACD and in the identification of concomitant ACD/IDA [26]. Mast et al. demonstrated 54 patients with hematologic and nonhematologic disease and iron status documented by bone marrow biopsy measurement of sTfR did not provide additional information to the measurement of serum ferritin [12]. In 120 adult anemic patients with chronic inflammation, chronic infection, or nonhematologic malignancy and anemia classification based on an examination of the bone marrow strain, Lee et al. demonstrated that sTfR is not superior to ferritin for detecting iron depletion [27]. These data are in agreement with those of Bultink et al. in 40 anemic RA patients with anemia classification based on bone marrow examination which showed that measurement of serum sTfR levels is not superior to the measurement of serum ferritin [14]. Similar results were found in studies with biochemically defined IDA and ACD [28, 29]. However, none of the mentioned studies evaluated the diagnostic performance of sTfR dependent on the presence of inflammation or the extent of inflammatory activity. Even studies including patients with chronic (rheumatic) inflammatory disease did not differentiate the diagnostic performance of sTfR dependent on the extent of inflammation operationalized as CRP- or ESR-levels.

The strength of our study was that it performed a differentiated evaluation of the added value of sTfR according to the inflammatory activity of the underlying rheumatic disease. Furthermore, in contrast to the above-mentioned studies, the diagnostic utility of sTfR was assessed by comparing it with the diagnostic performance of a combination of standard parameters of iron deficiency as the most effective use of traditional iron markers and reflecting the way conventional indices of iron status are used in clinical practice.

### 4.1. Study Limitations

Our study has limitations that should be taken into account.

First, in the absence of invasive bone marrow examination as a gold standard for the diagnosis of iron depletion, we were dependent on biochemical parameters for iron deficiency and inflammation to classify anemia groups. However, the classification was based on well-established algorithms described previously [8, 25–27].

Second, in this exploratory study, the stratification of patients into subjects with low and high inflammatory activity by means of the median CRP-level is somewhat arbitrary, and further studies aiming to define the range of inflammatory activity in RA or other inflammatory autoimmune diseases with an added value of sTfR measurement are required.

## 5. Conclusion

We conclude that for the detection of iron depletion, i.e., IDA or concomitant ACD and IDA, in anemic RA patients, the measurement of sTfR is superior to standard parameters of iron deficiency only in patients with high inflammatory activity, whereas in patients with low inflammation, the determination of sTfR does not exceed the diagnostic performance of standard parameters with even higher sensitivity of the combined use of standard markers of iron status.

## Figures and Tables

**Table 1:** Characteristics of patients under study. Data are given as means ± SD. Ranges are given in brackets and percentages in parentheses.

Number	116
Age, yr	69.3 ± 12.6 [29–92]
Women/men	57/57 (50/50)
Hb (g/dl), women	10.9 ± 1.0 [8.9–11.9]
Hb (g/dl), men	12.3 ± 1.4 [8.6–13.8]
Creatinine, mg/dl	0.92 ± 0.35 [0.47-2.34]
Autoantibodies	
Without any	53 (46)
RF	60 (52)
ACPA	58 (50)
RF and ACPA	54 (47)
Classification of anemia	
IDA	6 (5)
ACD	60 (52)
IDA/ACD	36 (31)
Other anemia	14 (12)
Indicators of disease activity	
CRP, mg/l	42.3 ± 49.1 [0.1-269.3]
ESR, mm/hour	46.6 (±31.0) [2–120]
DAS28 score	4.6 (±1.6) [0.8–9.2]
Indicators of iron status	
MCV, *μ*m^3^	88.0 (±6.1) [73–107]
MCH, pg	29.3 (±3.4) [23–35]
Ferritin, ng/ml	266.2 (±300.1) [10–1272]
Iron, *μ*g/dl	49.1 (±25.7) [10–118]
Transferrin g/l	1.3 (±0.6) [1.4–3.8]
TSAT, %	15.8 (±8.5) [2.9–62.2]
sTfR, mg/l	4.4 (±2.0) [2.2–13.6]
Immunosuppressive therapy	
None	29 (25.0)
Prednisolone mono low dose (≤7.5 mg/die)	26 (22.4)
csDMARD	41 (35.3)
bDMARD/tsDMARD	20 (17.2)
Anti-TNF	5 (4.3)
Abatacept	3 (2.6)
RTX	6 (5.2)
Anti-Il-6	4 (3.4)
JAK-I	2 (1.7)
Comorbidities	
Arterial hypertension	71 (61.2)
Cardiovascular disease (coronary heart disease, peripheral arterial disease, heart failure, and state after cerebral ischemia)	28 (24.1)
Absolute arrhythmia	20 (17.2)
Chronic renal insufficiency	13 (12.1)
Asthma	4 (3.4)
COPD	3 (2.6)
Hyperlipidemia	13 (12.1)
Diabetes mellitus type 2	20 (17.2)

Abbreviations: SD: standard deviation; Hb: hemoglobin; RF: rheumatoid factor; ACPA: anticitrullinated peptide antibody; IDA: iron deficiency anemia; ACD: anemia of chronic disease; CRP: C-reactive protein; ESR: erythrocyte sedimentation rate; DAS28: disease activity score in 28 joints; MCV: mean corpuscular volume; MCH: mean corpuscular hemoglobin; sTfR: soluble transferrin receptor; csDMARDs: conventional synthetic disease-modifying antirheumatic drugs; bDMARD: biologic disease-modifying antirheumatic drugs; tsDMARD: targeted synthetic disease-modifying antirheumatic drugs; RTX: rituximab; anti-Il-6: anti-interleukin-6 antibody; JAK-I: janus kinase inhibitor; COPD: chronic obstructive pulmonary disease.

**Table 2:** Sensitivity, specificity, and predictive values of sTfR and standard parameters of iron status alone and in combination for the diagnosis of IDA or ACD/IDA (*N* = 42) in the whole sample (*N* = 116).

	Sensitivity %	Specificity %	PPV %	NPV %
RBCI	23.8	95.9	76.6	69.1
Ferritin	54.8	86.5	69.5	77.0
Ferritin/RBCI	66.7	86.5	73.7	88.2
Ferritin/TSAT/RBCI	66.7	91.9	82.4	83.1
Ferritin/TSAT/CRP/RBCI	66.7	93.2	84.3	83.4
Ferritin/TSAT/CRP/sTfR/RBCI	54.8	95.9	89.9	82.6
sTfR	80.9	93.2	86.7	89.6

RBCI: red blood cell indices; IDA: iron deficiency anemia; ACD: anemia of chronic disease; ACD/IDA: anemia of chronic disease with concomitant true iron deficiency; TSAT: transferrin saturation; sTfR: soluble transferrin receptor.

**Table 3:** Sensitivity, specificity, and predictive values of sTfR and standard parameters of iron status alone and in combination for the diagnosis of IDA or ACD/IDA (*N* = 21) in patients with CRP ≤ median = 24.1 mg/l (*N* = 58).

	Sensitivity %	Specificity %	PPV %	NPV %
RBCI	19.0	100	100	68.7
Ferritin	76.2	78.4	66.6	85.3
Ferritin/RBCI	76.2	78.4	66.6	85.3
Ferritin/TSAT/RBCI	85.7	86.5	78.8	91.6
Ferritin/TSAT/CRP/RBCI	76.2	100	100	88.1
Ferritin/TSAT/CRP/sTfR/RBCI	57.1	100	100	80.5
sTfR	66.7	97.3	93.3	83.9

RBCI: red blood cell indices; IDA: iron deficiency anemia; ACD: anemia of chronic disease; ACD/IDA: anemia of chronic disease with concomitant true iron deficiency; TSAT: transferrin saturation; sTfR: soluble transferrin receptor.

**Table 4:** Sensitivity, specificity, and predictive values of sTfR and standard parameters of iron status alone and in combination for the diagnosis of IDA or ACD/IDA (*N* = 21) in patients with CRP > median = 24.1 mg/l (*N* = 58).

	Sensitivity %	Specificity %	PPV %	NPV %
RBCI	28.6	91.9	67.1	69.6
Ferritin	38.1	94.6	81.0	73.1
Ferritin/RBCI	52.4	89.2	72.7	76.8
Ferritin/TSAT/RBCI	61.9	91.9	81.3	81.1
Ferritin/TSAT/CRP/RBCI	52.4	94.6	84.4	77.7
Ferritin/TSAT/CRP/sTfR/RBCI	52.4	94.6	72.7	76.7
sTfR	100	89.9	83.6	100

RBCI: red blood cell indices; IDA: iron deficiency anemia; ACD: anemia of chronic disease; ACD/IDA: anemia of chronic disease with concomitant true iron deficiency; TSAT: transferrin saturation; sTfR: soluble transferrin receptor.

## Data Availability

The datasets used and/or analyzed during the current study are available from the corresponding author upon reasonable request.

## Additional Points

*Key Points*. (i) The position of sTfR in the diagnostic algorithm for iron deficiency in autoimmune inflammatory diseases is not defined. (ii) sTfR differentiates effectively between iron-depleted anemic states and anemia of chronic disease in patients with RA. (iii) For the detection of iron-depleted states, sTfR is superior to standard parameters of iron deficiency only in highly inflammatory states.

## Supplementary Materials

Supplementary Materials Supplementary Table S1: correlation coefficients (*r*) of sTfR with standard markers of iron deficiency. Supplementary Table S2: correlation coefficients (*r*) of iron deficiency parameters with inflammatory markers (CRP, ESR) and DAS 28.
