# Supplementary material for: Usefulness of Soluble Transferrin Receptor in the Diagnosis of Iron Deficiency Anemia in Rheumatoid Arthritis Patients in Clinical Practice
Source: Int J Rheumatol. 2022 Oct 12;2022:7067262. doi: 10.1155/2022/7067262 (PMC9581666; doi:10.1155/2022/7067262)
Supplement: Supplementary Materials — Supplementary Table S1: correlation coefficients (r) of sTfR with standard markers of iron deficiency. Supplementary Table S2: correlation coefficients (r) of iron deficiency parameters with inflammatory markers (CRP, ESR) and DAS 28. [file 7067262.f1.docx]

**Supplementary**

**Table S1** Correlation coefficients (Spearman`s rho, rs) of sTfR with standard markers of iron deficiency

|  | No. of cases (n) | Correlation coefficient (rs) | p value |
| --- | --- | --- | --- |
| Hb | 116 | -0.501 | <0.001 |
| Ferritin | 116 | -0.401 | <0.001 |
| Iron | 115 | -0.546 | <0.001 |
| Transferrin | 114 | -0.021 | 0.820 |
| TSAT | 114 | -0.604 | <0.001 |
| MCH | 116 | -0.615 | <0.001 |
| MCHC | 116 | -0.674 | <0.001 |
| MCV | 116 | -0.417 | <0.001 |

Abbreviations: sTfR, soluble transferrin receptor; Hb, hemoglobin; TSAT, transferrin saturation; MCH, mean corpuscular hemoglobin; MCHC mean corpuscular hemoglobin concentration; MCV, mean corpuscular volume.

**Table S2** Correlation coefficients (Spearman`s rho) of iron deficiency parameters with inflammatory markers (CRP, ESR) and DAS 28

|  | CRP | ESR | DAS 28 |
| --- | --- | --- | --- |
| sTfR | 0.279** | 0.265** | 0.306** |
| Ferritin | 0.386*** | 0.333*** | 0.134 |
| Iron | -0.583*** | -0.481*** | -0.418*** |
| Transferrin | -0.548*** | -0.487*** | -0.472*** |
| TSAT | -0.408*** | -0.344*** | -0.291** |
| MCH | -0.251* | -0.227* | -0.308** |
| MCHC | -0.152 | -0.153 | -0.235* |
| MCV | -0.279* | -0.231* | -0.315** |

Abbreviations: sTfR, soluble transferrin receptor; TSAT, transferrin saturation; MCH, mean corpuscular hemoglobin; MCHC mean corpuscular hemoglobin concentration; MCV, mean corpuscular volume; *p<0.05, **p<0.005, ***p<0.001
